# Diffraction phase microscopy imaging and multi-physics modeling of the nanoscale thermal expansion of a suspended resistor

**DOI:** 10.1038/s41598-017-04803-5

**Published:** 2017-07-04

**Authors:** Xiaozhen Wang, Tianjian Lu, Xin Yu, Jian-Ming Jin, Lynford L. Goddard

**Affiliations:** 1 Photonic Systems Laboratory, Department of Electrical and Computer Engineering, Micro and Nanotechnology Lab, University of Illinois at Urbana-Champaign, Urbana, Illinois 61801 USA; 2 Center for Computational Electromagnetics, Department of Electrical and Computer Engineering, University of Illinois at Urbana-Champaign, Urbana, Illinois 61801 USA

## Abstract

We studied the nanoscale thermal expansion of a suspended resistor both theoretically and experimentally and obtained consistent results. In the theoretical analysis, we used a three-dimensional coupled electrical-thermal-mechanical simulation and obtained the temperature and displacement field of the suspended resistor under a direct current (DC) input voltage. In the experiment, we recorded a sequence of images of the axial thermal expansion of the central bridge region of the suspended resistor at a rate of 1.8 frames/s by using epi-illumination diffraction phase microscopy (epi-DPM). This method accurately measured nanometer level relative height changes of the resistor in a temporally and spatially resolved manner. Upon application of a 2 V step in voltage, the resistor exhibited a steady-state increase in resistance of 1.14 Ω and in relative height of 3.5 nm, which agreed reasonably well with the predicted values of 1.08 Ω and 4.4 nm, respectively.

## Introduction

The dynamics of thermal-mechanical systems has received much attention in many different fields, such as physics, chemistry, and engineering. Many recent studies have focused on understanding the thermal response of micro- and nano-electro-mechanical systems (MEMS and NEMS) materials, devices and structures^1–8^, because thermal effects can significantly affect device performance when the power density is high. A representative example is the metal oxide semiconductor field-effect transistor (MOSFET). MOSFETs incorporated in modern integrated circuits (ICs) have been scaled down to tens of nanometers channel length. The International Technology Roadmap for Semiconductors (ITRS) predicts a 5 nm gate length device in 2019^9^. In addition, various micro- and nano-scale devices and structures such as ultraviolet nanowire lasers^2^, nanowire arrays^5^, carbon nanotubes^6^, and graphene-based NEMS devices^7^ have been developed for a broad range of applications including electronics, optoelectronics, thermoelectronics, electromechanics, and sensing. As the dimension of the devices is reduced, thermal characterization and modeling become critical in evaluating device performance and reliability under thermal stress. It is also important to understand the thermal behavior of the micro- and nano-scale devices and structures to develop novel materials related to thermal insulation and thermoelectric energy recovery. Therefore, micro- and nano-scale thermal measurement and modeling have become areas of intense research.

Compared to macroscopic thermal measurement, microscopic thermal characterization requires higher spatial resolution techniques. Scanning thermal microscopy (SThM), a promising scanning probe microscopy (SPM) based measurement technique, has been frequently used to investigate nanoscale thermal phenomena^10, 11^ over the past 20 years. Huber and co-workers investigated a 15 nm transient thermal expansion of a scanning tunneling microscope (STM) tip based on a combination of an atomic force microscope (AFM) and a laser as an external heating source^12^. A group led by Meriles and Riedo used a nitrogen-vacancy center in diamond attached to the apex of a silicon thermal tip as a local scanning temperature sensor^13^. They obtained nanometer-resolved thermal conductivity maps by combining magnetic resonance and AFM. These methods rely on built-in thermal sensors on STM or AFM probes. One challenge is the uncertainty of the heat conduction between the tip and substrate. Another challenge comes from the fabrication of a nanoscale tip and temperature sensor, which requires advanced nanofabrication techniques. Other nanoscale temperature measurement techniques, such as optical probing methods, have non-contact and non-destructive probing features. The near-field scanning optical microscopy (NSOM) methods, such as nanoscale fluorescence thermometry^14, 15^ and Raman thermometry^8, 16^, can successfully overcome the diffraction limit and produce an enhanced optical field within a very small region. However, the localized heating induced by the strong optical field can influence the temperature of the device under test or become destructive to the device. The aforementioned scanning methods also share a common limitation, namely the low speed in acquiring images over a large area.

Diffraction phase microscopy (DPM) is a real-time quantitative phase imaging (QPI) method^17^. As a single-shot non-destructive method, DPM can accurately monitor nanoscale dynamics *in*-*situ* ^18–25^. Epi-illumination diffraction phase microscopy (epi-DPM) provides a solution for reconstructing the three-dimensional (3D) shape of reflective structures. In this paper, we applied epi-DPM to characterize a suspended metal resistive bridge undergoing thermal expansion, with nanometer height measurement accuracy. This particular structure is interesting because of its application as a heating element for non-contact tuning of photonic membrane devices^26, 27^ and for actuating MEMS devices^28, 29^.

To understand the Joule-heating-induced thermal expansion, we used a coupled electrical-thermal-mechanical simulation. The coupled simulation consists of the electrical-thermal^30–33^ and the thermal-elastic simulations. In the electrical-thermal simulation, the dissipated power under a fixed direct current (DC) input voltage is calculated in the electrical analysis and is considered as the heat source in the thermal analysis. The electrical-thermal simulation is formulated in the transient regime such that both temporal and spatial profiles of temperature can be obtained. Because of temperature-dependent material properties, the electrical-thermal simulation deals with a nonlinear problem within each individual time step. The thermal-elastic simulation takes the steady-state temperature profile as input and calculates the displacement fields within a certain structure. It is necessary to have the coupled simulation formulated in 3D because both the lateral and vertical directions are critical paths of heat conduction in the suspended metal resistive bridge. The 3D coupled simulation is implemented based on the finite element method (FEM) because of FEM’s unmatched capability of modeling complex geometries and material properties^34^. It is worth mentioning that there are commercial software packages, e.g. COMSOL Multiphysics, that are capable of performing coupled simulations. However, we developed our own simulator because modeling the suspended bridge resistor requires fast, accurate, and memory-efficient multiscale analysis. The relative height change of the resistor is a few nanometers, whereas the top and bottom surfaces of the required simulation domain have an area approaching one square centimeter. Simulating the structure is especially challenging in 3D because of the enormous number of mesh elements. Despite the 96 GB of RAM available on our workstation, we were unsuccessful in performing the simulation using COMSOL Multiphysics because of the problem size.

In this paper, we fabricated a multilayer suspended resistor and studied its thermal properties under a DC input voltage. Understandings of the suspended resistor were also obtained through the coupled simulation, which provides the variations of parameters such as temperature, relative height change, dissipated power, and resistance under different applied voltages and the detailed 3D distributions of the temperature and displacement fields.

## Results

### Suspended resistor geometry and design

**Figure 1:**
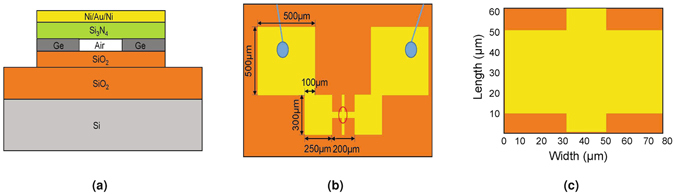
(**a**) Side view of the suspended resistor showing the material and device structure. The heights of Ni/Au/Ni, Si_3_N_4_, Ge, SiO_2_ and Si are 110 nm, 1000 nm, 80 nm, 1024 nm and 500 *μ*m, respectively. (**b**) Top view of the suspended resistor. The yellow area in the top view indicates the pattern of the Ni/Au/Ni layer and the orange area indicates that the SiO_2_ layer is exposed. (**c**) Zoomed-in view of the rectangular region inscribed in the red circle in (**b**). This rectangular area represents the field of view for the epi-DPM images.

As shown in Fig. 1, a multilayer suspended resistor was fabricated to have strong thermal expansion properties (Methods). Then, the sample was affixed to a printed circuit board (PCB) with double sided Scotch tape and electrical connections were made between the sample and the PCB by wire bonding. When a voltage is applied to the PCB, Joule-heating in the sample causes its temperature to increase. In order to simplify the analysis, we fabricated the suspended bridge with much larger (micrometer-size) lateral dimensions than its vertical dimensions (nanometer-size). Therefore, vertical expansion of the sample is expected to dominate.

### Mathematical modeling

**Figure 2:**
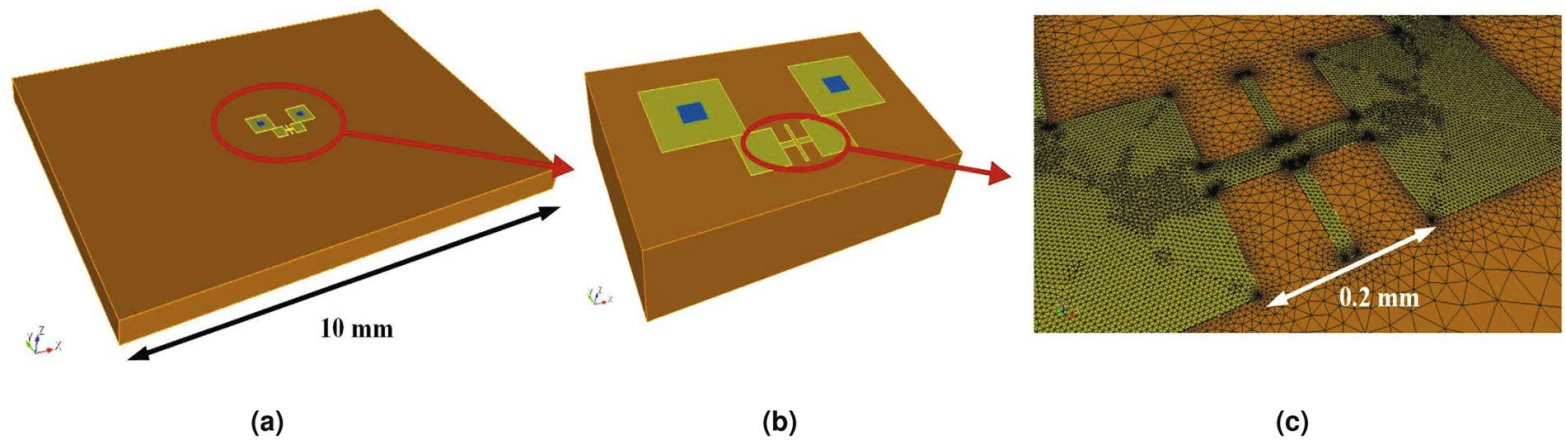
The 3D view of the geometry in the simulation and zoomed-in views showing the suspended resistor and its spatial discretization.

**Figure 3:**
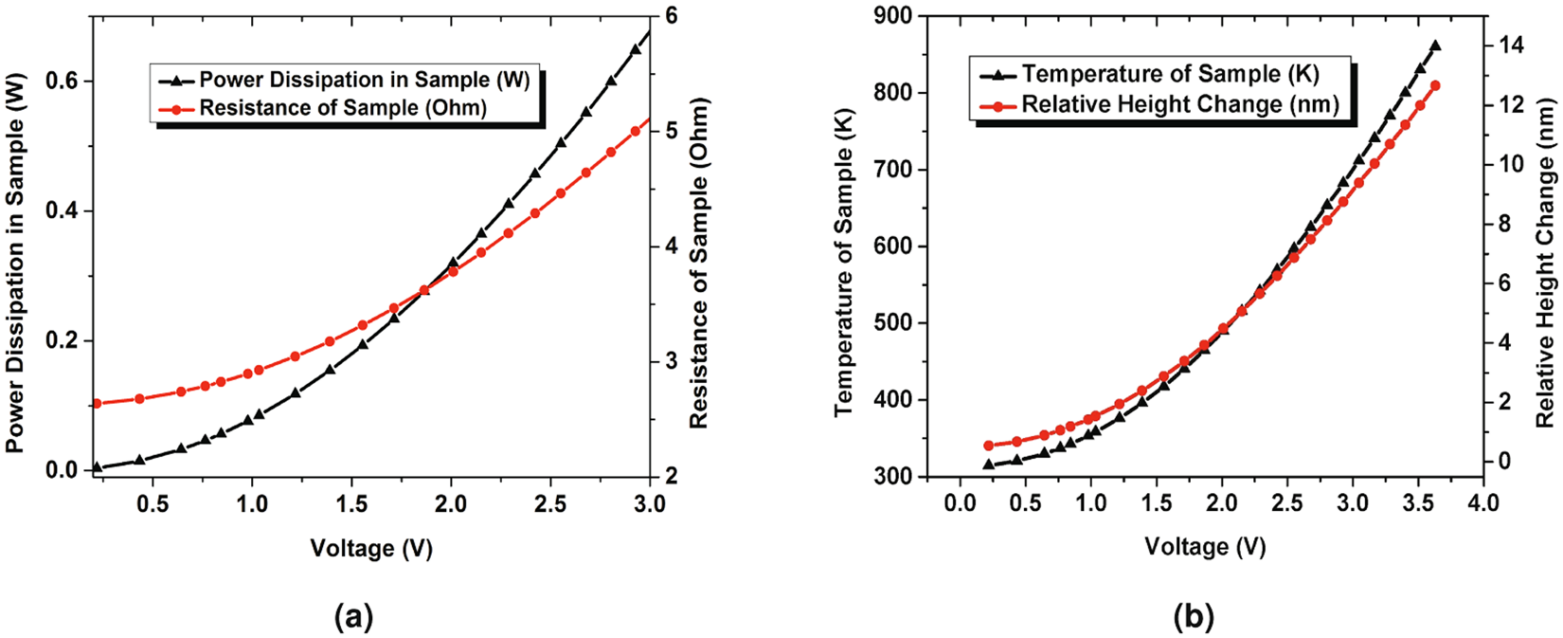
Simulated variations in the sample parameters versus voltage applied to the PCB include (**a**) power dissipation in the sample and resistance of the sample, and (**b**) temperature and relative height change. Note that because the PCB and bonding wires have 3 Ω of resistance, the power dissipated in the sample is about half the total power supplied.

Figure 2 shows the structure in the simulation with zoomed-in views of the suspended bridge and its spatial discretization. The entire simulation domain is 10 × 7 × 0.5 mm^3^. The zoomed-in portion at the suspended bridge has a dimension of 2 × 1.5 × 0.5 mm^3^. As shown in Fig. 3, the power dissipated in the sample increases and the temperature rises as the input voltage is increased. The increase in temperature creates a vertical deflection of the suspended bridge as is shown in Fig. 3(b). Because of the temperature-dependent resistivity of the gold layer, all the aforementioned quantities exhibit nonlinear variations with the input voltages.

**Figure 4:**
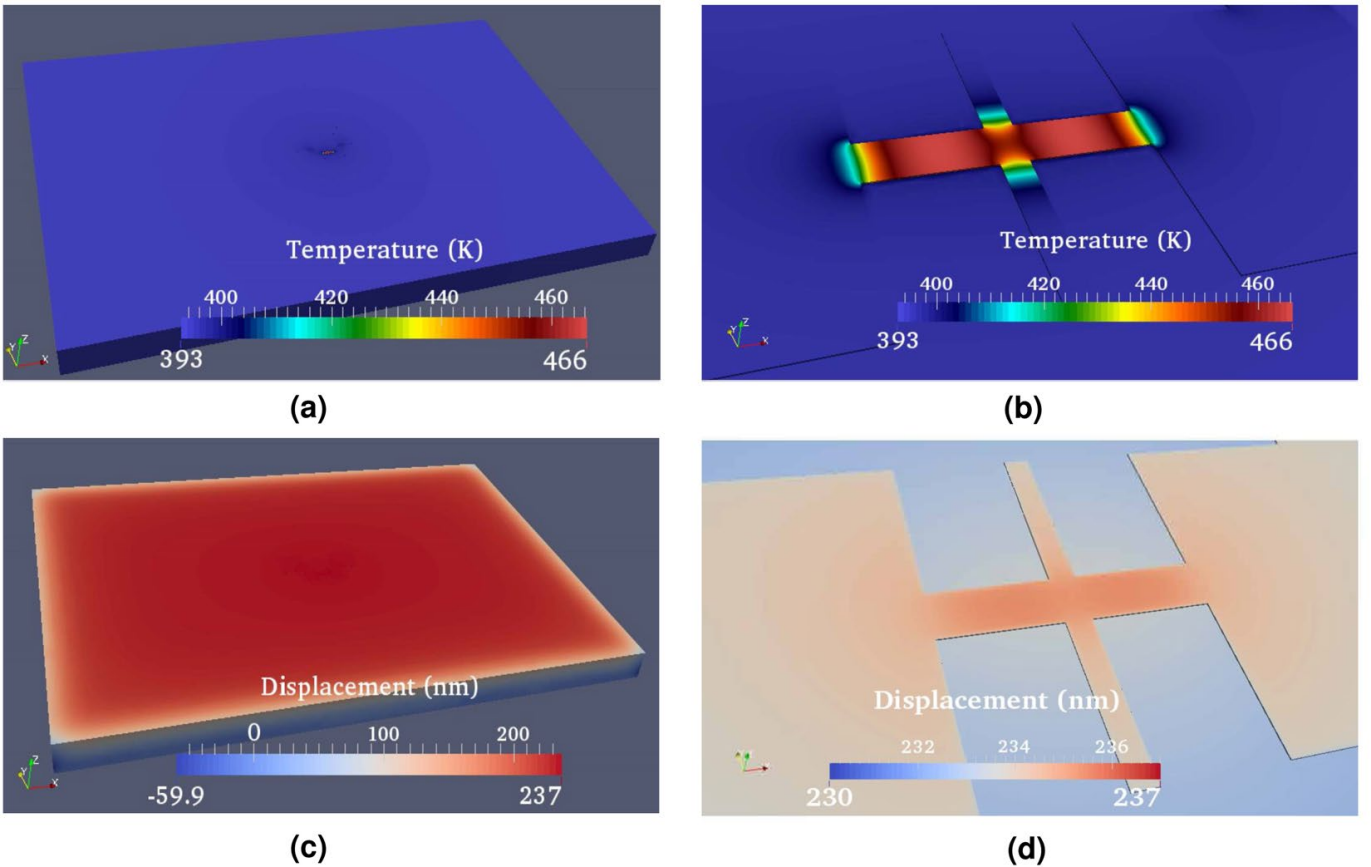
Simulation of (**a**) the steady-state temperature profile, (**b**) its zoomed-in view at the center of the suspended bridge of the resistor, (**c**) the distribution of the z-displacement profile, and (**d**) its zoomed-in view, under the input voltage of 2 V.

The detailed 3D distribution of the temperature obtained through the coupled simulation under the input of 2 V is provided in Fig. 4(a and b). Interestingly, because of the geometrical dimensions and material properties, the primary heat removal path is vertical conduction through the 80 nm thick air layer between the suspended resistor bridge and the oxide layer. An additional key heat path is laterally from the bridge to the side contact pads. The wings of the suspended resistor are a third path and explain why the temperature is not maximum at the very center of the bridge. Lateral heat conduction can be identified in Fig. 4(b) by finding places that have a temperature gradient.

The distribution for the vertical component of the displacement field is shown in Fig. 4(c and d). The bridge deflects upwards several nanometers because of the significant heating of the bridge. The oxide layer also heats up due to heat conduction from the bridge above and expands vertically. In the experiment, we measure the relative height change, i.e. the difference in these two deflections.

### Experimental setup and results

We used a conventional epi-DPM setup^19, 21^, including a 405 nm single mode fiber (SMF) coupled laser diode (QFLD-405-20S, QPhotonics), an ultra-violet (UV) transmission grating (300 lines/mm, 8.6° blaze angle), 4 f lens system, a 10 *μ*m diameter pinhole filter and a charge-coupled device (CCD) camera (Hamamatsu Orca ER C4742-80). The 405 nm laser is temperature controlled and electrically driven by a laser diode controller (ILX Lightwave LDC-3722), which enables a highly stable laser wavelength and low-noise output power. The beam is imaged at the sample plane by a microscope objective (Zeiss Plan-Apochromat 20X, NA 0.8) forming a field of view (FOV) of 81 × 62 *μ*m^2^. The reflected light goes through the grating, which separates the imaging field into multi-order copies. Only the first and zeroth orders are picked up by the pinhole filter and by the circular aperture, respectively, in the Fourier plane (FP); these orders served as the reference field and the image field, respectively. Compared with the previous DPM setup^21^, which had a visible transmission grating for 532 nm operation, we switched to the UV transmission grating because the diffraction efficiency of the zeroth and first orders increases from 4% and 47% to 26% and 65%, respectively. Therefore, the instrument noise per pixel is reduced from 3.0 nm to 2.0 nm. The exposure time is correspondingly reduced because of the higher intensity. Compared to our previous epi-DPM system^19, 21^, we exchanged the positions of the pinhole filter and the circular aperture and selected the stable zeroth order as the reference field. The first order then served as the image field. Because the sizes of the lenses are large, spherical aberrations are negligible. The more stable reference signal helped to further reduce the instrument noise per pixel from 2.0 nm to 1.5 nm. The details on the epi-DPM methods and phase retrieval are discussed in the previous works^19, 21, 35^.

The PCB with the sample mounted on it was fixed to the xyz translation stage of the DPM system. The resistance of the sample, bonding wires, and PCB was 5.6 Ω, of which 3.0 Ω was attributed to the bonding wires and PCB. Figure 1(b) shows the top view of the sample. The cross pattern was suspended and its two ends were connected to two rectangles (200 × 250 *μ*m^2^). Two bigger square pattern (500 × 500 *μ*m^2^) were used as contact pads for wire bonding. Gold wires were bonded between the sample and the PCB holes and the electric wires were soldered on the PCB holes to make connections to the electrical voltage source. The central bridge region of the resistor is observed by using a 20X objective and the observed FOV is shown in Fig. 1(c).

**Figure 5:**
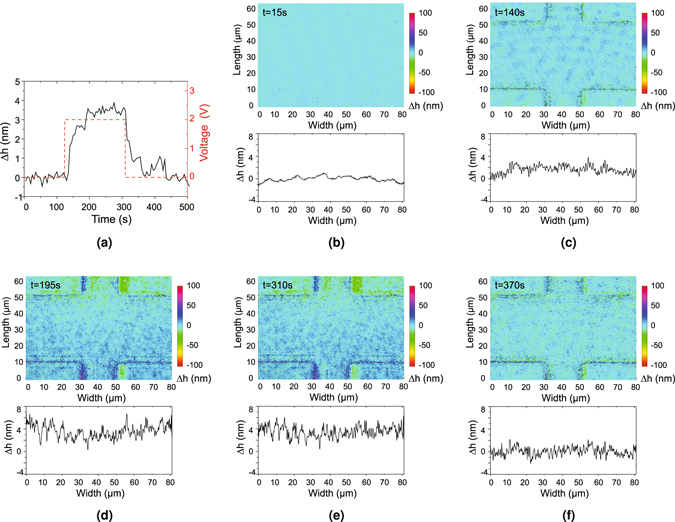
Relative height change measurements throughout the thermal expansion process. (**a**) Left axis: average relative height changes of a suspended resistor versus time; Right axis: applied voltage versus time. (**b**–**f**) Selected frames and their relative height changes at various times.

A background phase image was first collected without an applied voltage and subtracted from each subsequent phase image. This is commonly done with the DPM method to reduce the background noise^17–25^ and enables us to obtain an image sequence of the change in phase versus time. To reduce the propagation of phase errors during unwrapping, we temporally unwrapped the image sequence at each pixel^36^. Because this procedure directly results in the phase change at each pixel, spatial unwrapping is not needed. Epi-DPM phase images were collected at a rate of 1.8 frames/s. Phase images were converted to height images by multiplying by *λ*/4*π*, where *λ* is the wavelength of the light source. The height change of the central bridge of the suspended resistor, consisting of all six layers, was measured by averaging over the yellow area in Fig. 1(c), and the height change of the bottom two layers was measured by averaging around four corners, which are the orange areas in Fig. 1(c). Their difference, Δ*h*, which is defined as the relative height change, describes the height change of the top four layers. To further reduce the effect of temporal noise sources, these instantaneous relative height change images were averaged for 9 frames. As shown in Fig. 5(a), the relative height change of the suspended resistor is plotted as a function of time. The thermal expansion of the suspended resistor is thereby measurable with high accuracy.

Figure 5(a) shows the average relative height changes for the central bridge of the resistor as a function of time and the applied voltage as a function of time. After application of the 2 V voltage pulse, the sample started to expand and its relative height increased from t = 140 s to t = 195 s. At t = 140 s, we obtained a relative height change of 1.4 nm, which was defined as the initial relative height change. Then, the entire sample including the substrate reached thermal steady state between t = 195 s and t = 310 s and the relative height change became maximum. The average relative height change during this period was 3.5 nm, which agreed with the predicted value of 4.4 nm as shown in Fig. 3(b). After that, the voltage supply was turned off. The sample gradually returned to its initial state and its corresponding relative height change decreased from t = 315 s to t = 360 s. Figure 5(b–f) present the frames and their relative height change curves at five key moments: before expansion (t = 15 s), at the beginning of expansion (t = 140 s, i.e. at thermal steady state for the suspended bridge), at the thermal steady state for the substrate (t = 195 s), at the beginning of recovery (t = 310 s), and after the recovery (t = 370 s). From these frames, we find that the thermal expansion process of a suspended resistor can be reconstructed using epi-DPM and the coupled model can predict the height change when the suspended bridge is in thermal steady state.

**Figure 6:**
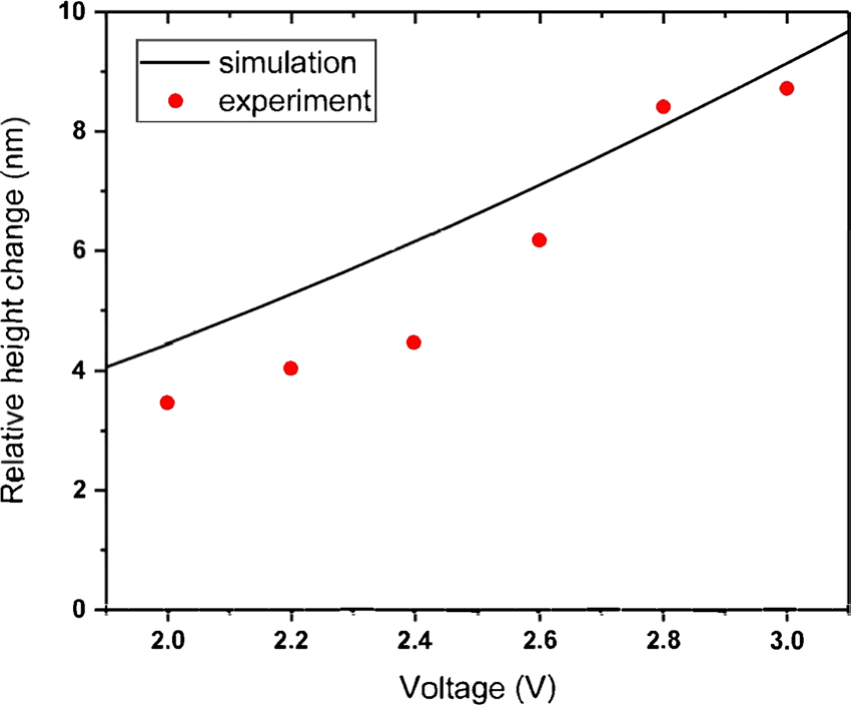
Steady-state relative height changes versus input voltage.

In order to quantify the dependency of thermal expansion on voltage, the relative height change measurement was repeated for various DC voltages. As shown in Fig. 6, the steady-state relative height changes for both theory and experiment are plotted as a function of applied voltage. The relative height change increases rapidly as the applied voltage increases.

**Figure 7:**
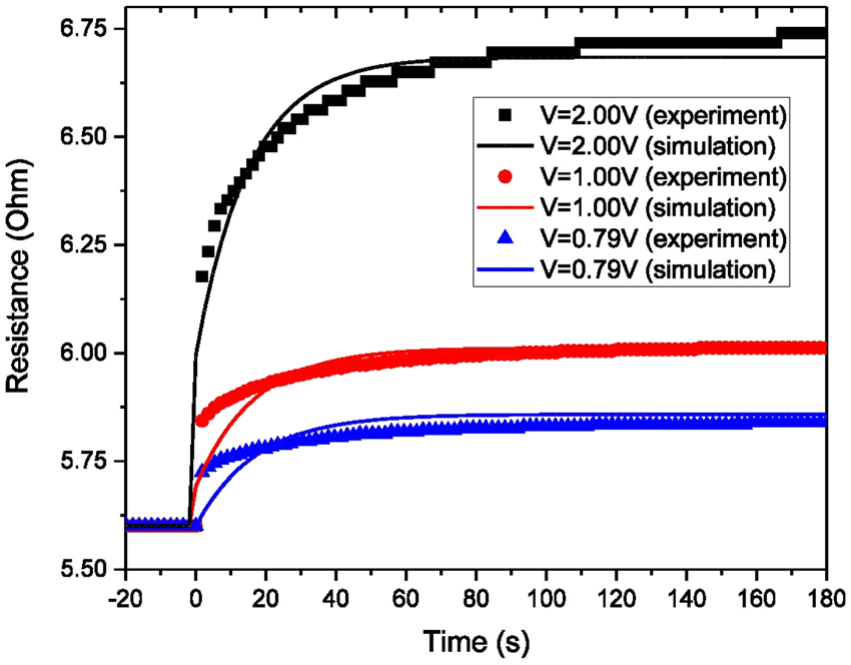
Simulation and experimental results of the resistance of the sample, bonding wires, and PCB versus time.

To further confirm the accuracy of our coupled model, the combined resistance of the sample, bonding wires, and PCB was also measured during the thermal expansion process. Figure 7 shows how the resistance increases as a function of time. Three different DC voltages were applied. For t < 0 s, we plotted the resistance of the sample, bonding wires, and PCB without heating. This value was 5.6 Ω. At t = 0 s, the voltage was applied and the resistance increased almost instantly. Then, the resistance increased slowly and became stable in the last 60 s. The initial jump in resistance is attributed to the heating of the suspended bridge itself and the slow increase in resistance is attributed to the gradual heat spreading across the large area substrate. The simulation results match well with the experimental results.

## Discussion

We applied epi-DPM to observe the nanoscale thermal expansion of a suspended bridge resistor. We quantified the relative height change with sub-nanometer accuracy. The results show that even under a low dissipated power of a few hundred milliwatts, there can be several nanometers of relative expansion. In some nanoscale device applications, this expansion can be a source for performance degradation or complete device failure.

We must point out that choosing appropriate boundary conditions that faithfully represent the experimental conditions is critical for accurate simulation results. For example, although modeling only the zoomed-in region of the sample near the suspended resistor is adequate for capturing the fast dynamics of the initial jump in resistance, this approach fails to capture the slower dynamics caused by substrate heating. Another related example is that a large thermally conductive mass (e.g. the substrate on the PCB) is often approximated by an isothermal boundary. However, the presence of tape between the substrate and the PCB creates an adiabatic thermal boundary condition there because the tape is thick and has a very low thermal conductivity. Including an isothermal boundary in the simulation domain can erroneously reduce the time scale for the expansion dynamics by a few orders of magnitude.

In conclusion, we presented a coupled electrical-thermal-mechanical model to understand the underlying physics and were able to predict the 3D temperature profile, the change in resistance, and the relative height expansion under different applied voltages. The simulated and experimental results for both the thermal expansion and the change in resistance showed good agreement. With the growing interest in predicting and engineering the coupled dynamics of micro- and nano-scale systems, we anticipate that this model and measurement technique will have many applications.

## Methods

### Sample preparation

As shown in Fig. 1(a), a suspended resistor was fabricated on a 500 *μ*m thick Si substrate with a 1024 nm thick SiO_2_ insulator layer. The resistor consists of a 110 nm thick nickel/gold/nickel layer that was deposited on a low stress 1000 nm thick SiN_*x*_ film that sits on an 80 nm thick germanium sacrificial layer. Traditional fluoric dry etching was used to define the suspended bridge pattern. Once the sacrificial layer was selectively undercut by an XeF_2_ etchant, the resistor was released from the substrate and formed the suspended structure. Next, electrical connections were made to a PCB by wire bonding the large area contact pads shown in Fig. 1(b) to corresponding pads on the PCB.

### Physical mechanism

The governing equations of the electrical-thermal simulation^32^ include the current continuity equation

1 $$\nabla \cdot (\vec{J}+\frac{\partial \vec{D}}{\partial t})=0$$

and the heat conduction equation based on conservation of energy

2 $$\rho {c}_{{\rm{p}}}\frac{\partial T}{\partial t}=\nabla \cdot (\kappa \nabla T)+q,$$

where $\vec{J}$ represents the conduction current density, $\vec{D}$ denotes the electric displacement field, *T* denotes the temperature, *ρ* represents the mass density, *c*_*p*_ is the specific heat capacity, and *κ* denotes the thermal conductivity. The rate of volumetric heat generation *q* in equation (2) is associated with the volumetric power density dissipated in the gold layer, which can be written as

3 $$\frac{{P}_{{\rm{Joule}}}}{V}=\sigma {|\vec{E}|}^{2}.$$

where *V* is the volume, *σ* is electrical conductivity and $\vec{E}$ denotes the electric field. Convection heat transfer is described through the boundary condition

4 $$\hat{n}\cdot (\kappa \nabla T)=-h(T-{T}_{{\rm{a}}}),$$

where $\hat{n}$ is a unit normal vector pointing outward to the surface, *h* is the convection heat transfer coefficient, and *T*_*a*_ denotes the ambient temperature. Radiation heat transfer is also taken into account through a boundary condition, which can be written as

5 $$\hat{n}\cdot (\kappa \nabla T)=-{\varepsilon }_{{\rm{rad}}}{\sigma }_{{\rm{rad}}}({T}^{4}-{T}_{{\rm{a}}}^{4}),$$

where ${\varepsilon }_{{\rm{rad}}}$ is the surface emissivity and *σ*_rad_ is the Stefan-Boltzmann constant.

The resistivity of gold is temperature dependent

6 $${\rho }_{{\rm{Au}}}={\rho }_{{\rm{Au}}0}[1+{\alpha }_{{\rm{T}}}(T-{T}_{0})],$$

where ${\rho }_{{\rm{Au}}0}=2.44\times {10}^{-8}\,{\rm{\Omega }}\cdot {\rm{m}}$ is the resistivity of gold at reference temperature *T*_0_ = 293 K and *α*_T_ = 0.0034 K^−1^ is the temperature coefficient of the resistivity. The Si_3_N_4_, air, and SiO_2_ layers are treated as perfect electrical insulators. The thermal conductivity of the silicon substrate is temperature dependent^37^, which can be expressed as

7 $${\kappa }_{{\rm{Si}}}=2.475\times {10}^{5}\,{T}^{-1.3}\quad \mathrm{(273}\,{\rm{K}}\le T\le 1000\,{\rm{K}}).$$

The governing equations of the thermal-elastic simulation^38^ consist of the conservation of momentum

8 $$\rho \frac{\partial {v}_{i}}{\partial t}=\frac{\partial {\sigma }_{ij}}{\partial {x}_{j}}+{f}_{i},$$

the infinitesimal strain-displacement relations

9 $${\varepsilon }_{ij}=\frac{1}{2}(\frac{\partial {u}_{i}}{\partial {x}_{j}}+\frac{\partial {u}_{j}}{\partial {x}_{i}}),$$

and the constitutive equation

10 $${\sigma }_{ij}={C}_{ijkl}({\varepsilon }_{kl}-\alpha {\delta }_{ij}{\rm{\Delta }}T),$$

where *u*_*i*_, *v*_*i*_, *f*_*i*_ represent the components of the displacement vector, the velocity vector, and the vector of body force per unit volume, respectively, and *x*_*i*_ (*i* = *x*, *y*, *z*) are the space coordinates, *C*_*ijkl*_ denotes the stiffness coefficient, *α* is the thermal expansion coefficient, Δ*T* is the temperature change from a reference temperature (room temperature in this work) at which the structure is regarded as free of stress, and ${\varepsilon }_{ij}$ and *σ*_*ij*_ denote the components of the strain and stress tensors, respectively. Note that a steady-state assumption is made. Consequently, the left-hand side of equation (8) becomes zero. In the thermal-elastic simulation, the displacement fields are calculated at thermal steady state when the temperature distribution stabilizes. Combining these three equations yields the governing equation for the thermal-stress analysis

11 $$\frac{1}{2}\frac{\partial }{\partial {x}_{j}}[{C}_{ijkl}(\frac{\partial {u}_{k}}{\partial {x}_{l}}+\frac{\partial {u}_{l}}{\partial {x}_{k}})]=-\frac{\partial }{\partial {x}_{j}}({C}_{ijkl}\alpha {\delta }_{kl}{\rm{\Delta }}T)-{f}_{i}.$$

The simulation volume includes the sample and the air above it. A mixed boundary condition consisting of convection and radiation is applied at the upper sample-air interface. The surface emissivity of SiO_2_ in equation (5) is set to be 0.7 and the effective heat transfer coefficient in equation (4) is set to be 30 W m^−1^ K^−1^. A convection boundary condition is also applied to the four sides of the sample-air interface. An adiabatic boundary condition is used on the bottom sample interface with the tape. The material properties used in the simulation are shown in Table 1. Because of the large dimension of the silicon substrate and the large variation of its thermal conductivity in the temperature range between 300 K and 500 K, the thermal conductivity model of the silicon substrate plays a critical role in the transient thermal response. This is the reason that we need to use a temperature-dependent model in our simulation. There is excellent agreement between theory and experiment in the steady state values of the relative height change and resistance shown in Figs. 6 and 7, respectively. We believe that the small differences in the time scale of the initial resistance jumps in Fig. 7 is because the model does not include heating effects in the bonding wires.

**Table 1:** Material properties in the simulation.

	Thermal conductivity (W m^−1^ K^−1^)	Density (kg m^−3^)	Heat capacity (J kg^−1^ K^−1^)	Thermal expansion coefficient (10^−6^ K^−1^)	Young’s modulus (GPa)	Poisson’s ratio
Au	317	19.3 × 10^3^	129	14.2	70	0.46
Si_3_N_4_	20	3.1 × 10^3^	700	2.3	250	0.26
Ge	58	5.3 × 10^3^	310	5.9	103	0.28
SiO_2_	1.4	2.2 × 10^3^	730	0.5	70	0.17
Si	equation (7)	2.3 × 10^3^	500	2.6	170	0.28
Air	0.024	1.1	1009	0.0	103	0.26
